# Integrative Approach to Pain Genetics Identifies Pain Sensitivity Loci across Diseases

**DOI:** 10.1371/journal.pcbi.1002538

**Published:** 2012-06-07

**Authors:** David Ruau, Joel T. Dudley, Rong Chen, Nicholas G. Phillips, Gary E. Swan, Laura C. Lazzeroni, J. David Clark, Atul J. Butte, Martin S. Angst

**Affiliations:** 1 Department of Anesthesia, Stanford University School of Medicine, Stanford, California, United States of America; 2 Division of System Medicine, Department of Pediatrics, Stanford University School of Medicine, Stanford, California, United States of America; 3 Department of Anesthesia and Pain Management, Veterans Affairs Palo Alto Health Care System, Palo Alto, California, United States of America; 4 Center for Health Sciences, SRI International, Menlo Park, California, United States of America; 5 Departments of Psychiatry and Behavioral Sciences and of Pediatrics, Stanford University School of Medicine, Stanford, California, United States of America; University of Chicago, United States of America

## Abstract

Identifying human genes relevant for the processing of pain requires difficult-to-conduct and expensive large-scale clinical trials. Here, we examine a novel integrative paradigm for data-driven discovery of pain gene candidates, taking advantage of the vast amount of existing disease-related clinical literature and gene expression microarray data stored in large international repositories. First, thousands of diseases were ranked according to a disease-specific pain index (DSPI), derived from Medical Subject Heading (MESH) annotations in MEDLINE. Second, gene expression profiles of 121 of these human diseases were obtained from public sources. Third, genes with expression variation significantly correlated with DSPI across diseases were selected as candidate pain genes. Finally, selected candidate pain genes were genotyped in an independent human cohort and prospectively evaluated for significant association between variants and measures of pain sensitivity. The strongest signal was with rs4512126 (5q32, *ABLIM3*, P = 1.3×10^−10^) for the sensitivity to cold pressor pain in males, but not in females. Significant associations were also observed with rs12548828, rs7826700 and rs1075791 on 8q22.2 within *NCALD* (P = 1.7×10^−4^, 1.8×10^−4^, and 2.2×10^−4^ respectively). Our results demonstrate the utility of a novel paradigm that integrates publicly available disease-specific gene expression data with clinical data curated from MEDLINE to facilitate the discovery of pain-relevant genes. This data-derived list of pain gene candidates enables additional focused and efficient biological studies validating additional candidates.

## Author Summary

The mechanisms underlying pain are incompletely understood, and are hard to study due to the subjective and complex nature of pain. From a genetics perspective, the discovery of genes relevant for the processing of pain in humans has been slow and genome-wide association studies have not been successful in yielding significantly associated variants. Targeted approaches examining specific candidate genes may be more promising. We present a novel integrative approach that combines publicly available molecular data and automatically extracted knowledge regarding pain contained in the literature to assist the discovery of novel pain genes. We prospectively validated this approach by demonstrating a significant association between several newly identified pain gene candidates and sensitivity to cold pressor pain.

## Introduction

A significant number of diseases are associated with pain, thereby affecting the quality of life of many individuals. The Institute of Medicine's recent report titled, “Relieving Pain in America” presented pain as a public health challenge, and emphasized the need for an integrative approach to understand mechanisms underlying pain [1]. Such understanding is critical for developing more effective and individualized strategies targeting the prevention and treatment of pain.

Studies in rodents and humans have established the importance of genetic factors in the processing of pain [2], [3], [4]. However, identifying genes important to complex phenotypes such as pain using genome-wide association studies has been challenging [5]. Candidate gene studies have identified many gene variants associated with susceptibility to pain [6], [7]. Despite these advances, genetic discoveries in the domain of pain have been slow in forthcoming compared to other fields [8].

Pain is among the most difficult phenotypes to study due to its complex and subjective nature. The perception of pain is influenced by a multitude of variables including gender, age, mood, ethnicity and genetic factors [9], and a recent meta-analysis highlighted the overall small effect size attributable to any gene variant associated with the processing of pain [10]. The polygenetic nature of pain and the small effect size of gene variants pose significant challenges for pain gene discovery.

Candidate gene studies have proven successful in the identification of pain genes. A particularly promising approach used gene expression microarray analysis to select candidate genes [11], [12]. More recently a meta-analysis of publicly available microarray data from rodents exposed to neuropathic or inflammatory pain was able to efficiently prioritize pain-related genes [13]. A similar approach using human gene expression data could be highly beneficial in generating data-driven hypotheses for pain genetics. However, there is currently a paucity of public gene expression data related to specific human pain conditions.

In this study, we describe an integrative approach exploiting publically available gene expression data for a large set of disease conditions to develop a disease-specific pain index (DSPI). This approach is based on the hypothesis that differences at the gene expression level correlating with pain indices would allow identifying novel pain gene candidates [14]. We validated this approach through a targeted genetic association study in an independent human cohort, where variants of selected pain gene candidates were evaluated for associations with experimental pain sensitivity measures in humans.

## Results

### Disease-specific pain index

We built the disease-specific pain index (DSPI) using our literature-based approach, in which 2962 diseases were ranked according to their disease-pain ratio. Table 1 displays the 20 diseases with the highest pain indices. Diseases included Prinzmetal's angina, neuralgia, causalgia, chronic plantar fasciitis and polyarthralgia; all conditions associated with severe pain. Among the diseases ranked at the bottom of the pain index list were fetal alcohol syndrome, cretinism, hermaphroditism and fetal erythroblastosis; all conditions not primarily associated with pain (Table S1). Inspection of the DSPI indicates that diseases with a high pain index are typically associated with significant clinical pain, while pain is not a hallmark of diseases with a low pain index. As such, the pain index captures relevant aspects of disease-related pain. However, the DSPI relies on the fraction of disease-related publications in PubMed that are associated with the Medical Subject Heading (MESH) term “pain” and therefore, is subject to some bias. For example, a disease associated with significant pain but hardly studied in the context of pain would rank inappropriately low on the DSPI list. A practical example is the pain index of cholera with a rank of 2936, which is at the bottom of the list. Cholera is clearly associated with painful symptoms. Inspection of the DSPI generally revealed relatively low ranks for infectious diseases, likely indicating that the research community predominantly focuses on the most relevant aspects of the condition under study. This suggests that the DSPI also captures to some extend the relevance of pain across multiple diseases.

**Table 1:** The 20 diseases with the highest disease-pain ratio from the DSPI are listed out of a total of 2962 diseases.

Diseases	Ratio
Prinzmetal's Angina	1
Unstable Angina	1
Vertebrogenic Pain Syndrome	1
Pain, Menstrual	1
Neuralgia, Atypical	1
Mechanical Low Back Pain	1
Polyarthralgia	1
Somatic Sensation Disorders	1
Postherpetic Neuralgia	1
Failed Back Surgery Syndrome	1
Piriformis syndrome	1
Slit Ventricule Syndrome	1
Causalgia Syndrome	0.62
Vulvar Vestibulitis	0.52
Pseudomelia	0.48
Myofascial Pain Syndromes	0.47
Chronic Plantar Fasciitis	0.45
Perineural Cysts	0.43
Craniomandibular Diseases	0.43
Vulvodynia	0.42

The ratio indicates the number of disease citations in PubMed associated with the MeSH term “pain” in relation to the total number of diseases citations.

### Identification of candidate pain genes

As previously described, the raw microarray data for 311 diseases were extracted from public gene expression databases [15], [16], [17]. A list of 3812 differentially expressed (DE) genes was then compiled (see Materials and Methods). Pain indices were available for 121 of the 311 diseases with suitable microarray data. The 121 disease-related gene expression changes were ordered according to the DSPI. For each of the 3812 differentially expressed genes, the gene expression fold change across every disease was correlated with the DSPI. This allowed identifying genes whose expression changes were significantly correlated with pain.

**Figure 1:**
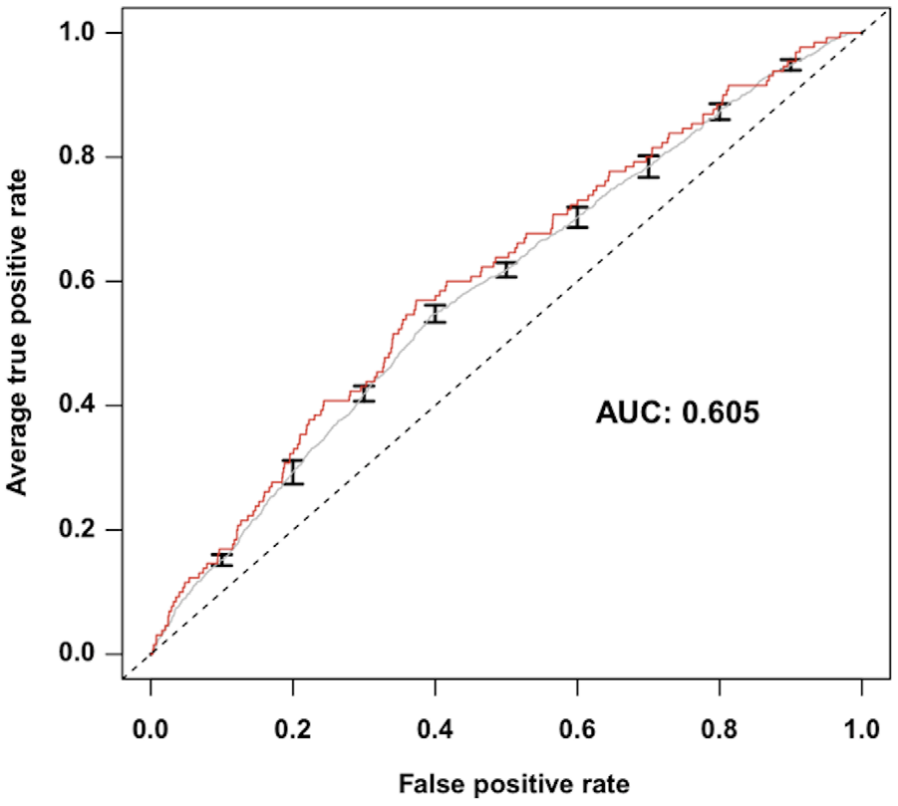
Receiver Operating Characteristic (ROC) curve. The ROC curve depicts the performance of our algorithm to identify known pain genes listed in the Pain Genes Database [18]. The area under the ROC curve is 60.5%, which is significantly different from random chance. Maximum sensitivity and specificity are 56.9% and 62.7% respectively.

The sensitivity and accuracy of this strategy for capturing genes implicated in the processing of pain was first evaluated with the aid of the Pain Gene Database (PGD) [18]. The PGD catalogs genes whose transgenic or knockout mouse counterparts have exhibited changes in pain-related phenotypes. The PGD is actively maintained and, to our knowledge, is the only pain-related gene database. Figure 1 shows the receiver operating characteristic (ROC) curve with confidence intervals. The area under the curve (AUC) was 60.5% indicating a prioritization of known pain genes from the PGD by our method.

**Figure 2:**
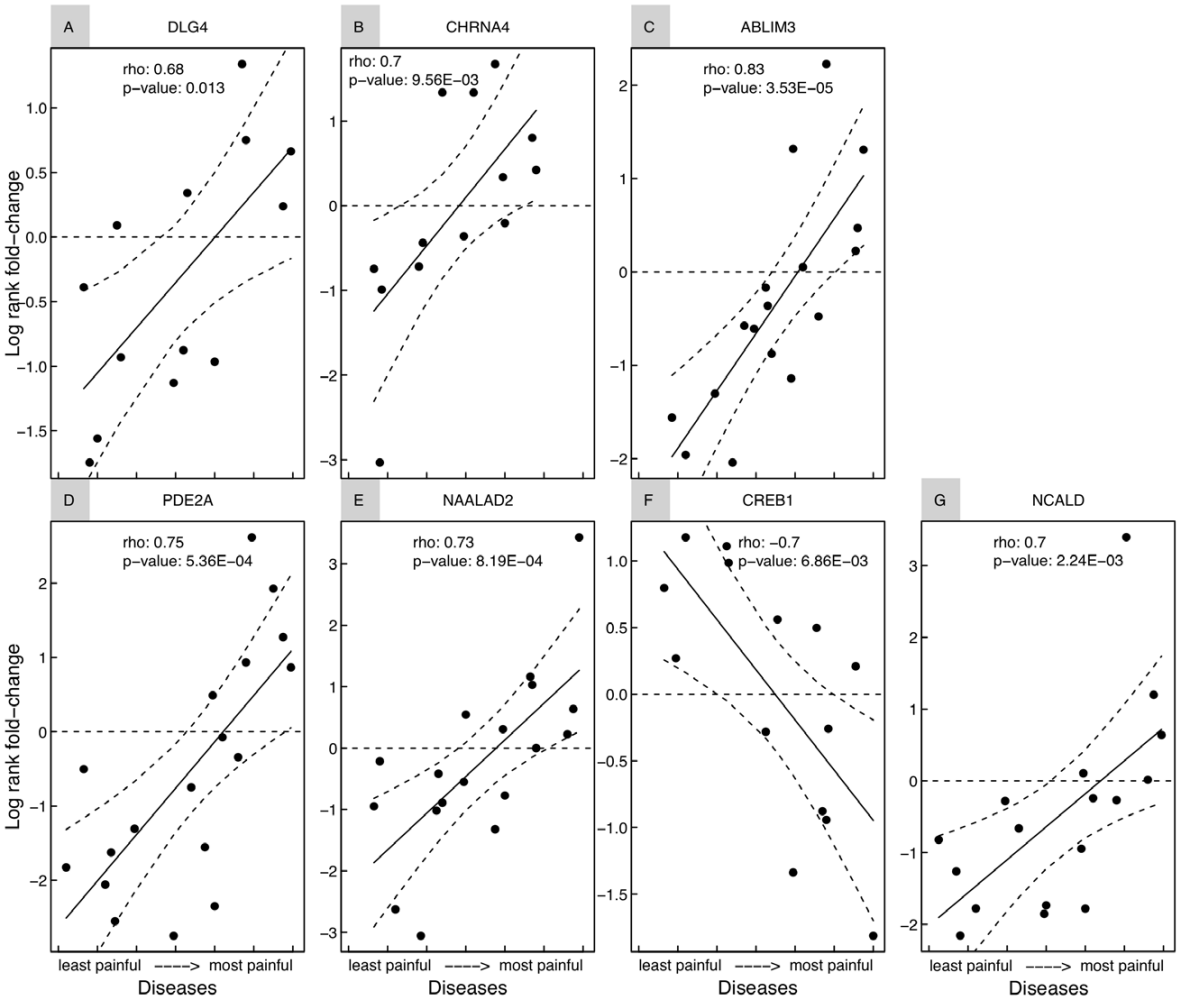
Genes with significant correlation between expression rank fold-change and disease-specific pain index (DSPI). (A, B) *DLG4* and *CHRNA4* are known pain genes listed in the Pain Gene Database. (C–G) *ABLIM3*, *PDE2A*, *NAALAD2*, *CREB1* and *NCALD* were selected for further investigation through genotyping in an independent human cohort. X-axis represents disease ordered according to the DSPI. Y-axis displays the rank fold change. Solid line indicates linear regression fit. Rho Spearman correlation coefficient and uncorrected correlation p-values are shown. The curved dashed line represents the 95% confidence interval of the linear regression performed against the DSPI.

We evaluated the significance of the association of the 3812 genes with the DSPI using a threshold-based estimated false discovery rate. Forty-seven genes were significantly associated with the DSPI (pFDR<0.01; Table 2). Among the 47 genes, two genes, *DLG4* (*PSD-95*) and *CHRNA4*, were referenced in the PGD [19], [20]. *DLG4* and *CHRNA4* were both found to have expression changes in 13 of 121 diseases that were positively correlated with pain indices (Figure 2A–B).

**Table 2:** Forty-seven genes showed expression changes that were significantly correlated with the DSPI.

A. Gene positively correlated with painful diseases			B. Genes negatively correlated with painful diseases		
Gene Symbol	correlation	pFDR	Gene Symbol	correlation	pFDR
**ABLIM3**	0.83	2.28E-04	**ZNF131**	−0.63	9.68E-03
**EIF1**	0.78	6.66E-04	**KRR1**	−0.63	9.68E-03
**CHST15**	0.78	7.67E-04	**TTN**	−0.63	9.61E-03
**PDE2A**	0.75	1.45E-03	**MYLIP**	−0.64	9.45E-03
**MED6**	0.74	1.76E-03	**ACTR2**	−0.64	9.18E-03
**NAALAD2**	0.73	2.00E-03	**NUMA1**	−0.64	8.10E-03
**SEZ6L**	0.73	2.18E-03	**STAT2**	−0.65	7.91E-03
**AOX1**	0.72	2.54E-03	**APOA1**	−0.65	7.32E-03
**CRY2**	0.71	2.85E-03	**ADD1**	−0.65	7.03E-03
**TRIM29**	0.71	3.16E-03	**ITPKB**	−0.66	6.34E-03
**NCALD**	0.70	3.27E-03	**TNFSF11**	−0.68	4.82E-03
**CHRNA4**	0.70	3.38E-03	**SRP72**	−0.69	4.43E-03
**KCTD13**	0.70	3.68E-03	**TRAF4**	−0.69	4.28E-03
**CCNO**	0.69	3.84E-03	**AKIRIN1**	−0.69	4.06E-03
**RPS11**	0.69	4.06E-03	**CREB1**	−0.70	3.46E-03
**DHRS2**	0.69	4.06E-03	**HSP90B1**	−0.73	2.11E-03
**DLG4**	0.68	4.82E-03	**MARCKSL1**	−0.74	1.89E-03
**REG1A**	0.68	4.82E-03	**MTDH**	−0.76	1.22E-03
**ZFPM2**	0.68	5.02E-03	**LUC7L3**	−0.77	8.96E-04
**RFTN1**	0.67	5.75E-03			
**PTPRK**	0.67	5.75E-03			
**GAP43**	0.66	6.45E-03			
**MAPK8IP3**	0.65	7.03E-03			
**GP5**	0.65	7.91E-03			
**CNTN3**	0.64	8.16E-03			
**ANKRD7**	0.64	8.29E-03			
**C2orf40**	0.64	8.71E-03			
**TAL1**	0.64	9.15E-03			

In light of this significant but modest prioritization of pain related genes, we applied our pipeline to another medically relevant concept: “Inflammation”. As described above for pain we extracted from MEDLINE a Disease-Specific Inflammation Index (DSII) and retrieved gene significantly associated with this index. Using genes belonging to the Gene Ontology category “Inflammatory Response” (GO:0006954) as gold standard we computed the area under the ROC curve. Figure S1 shows a clear prioritization of known “Inflammatory response” genes through our pipeline with an AUC of 73.2%.

### Polymorphisms in *ABLIM3* and *NCALD* are associated with cold pain threshold

Selected genes from the candidate list were prospectively tested for variants that may be associated with differential pain sensitivity in an independent human cohort. These genes were chosen based on their high correlation with the DSPI and plausible biology as assessed by the available literature and human expression profile across tissue using The Scripps Research Institute BioGPS database [21]. The selected genes were: (i) *ABLIM3* (actin binding LIM protein family, member 3), *PDE2A* (phosphodiesterase 2A, cGMP-stimulated), *CREB1* (cAMP responsive element binding protein 1), *NAALAD2* (N-acetylated alpha-linked acidic dipeptidase 2), and *NCALD* (neurocalcin delta) (Figure 2C–G).

*ABLIM3* was selected as our top candidate as it showed the highest correlation with the DSPI. The cGMP-sensitive phosphodiesterase *PDE2A* localizes at the neuronal membrane in synapses and has been described as being regulated by TNFα, a known proinflammatory cytokine shown to sensitize primary nociceptors [22]. Additionally, a recent study on Grueneberg ganglion neurons, that are proposed thermosensors, revealed a key role of cGMP enzyme in cold temperature sensing [23]. Interestingly, *PDE2A* provides a mechanism for nitric oxide-mediated cGMP synthesis to control intracellular concentrations of cAMP [24]. cAMP is a key second messenger that activate numerous downstream protein, notably cyclic-AMP-response element (CRE)-binding protein (CREB) that activate classical immediate-early genes such as *c-Fos*, which are associated with nociceptive afferent activation [25], [26]. *NAALAD2* is highly similar in sequence to *NAALAD1* and both hydrolyze N-acetyl-L-aspartate-L-glutamate (NAAG) to N-acetyl-aspartate and glutamate, a neuropeptide that activates and antagonizes neuronal N-methyl-D-aspartate (NMDA) receptors [27]. Based on nociceptive tests in rats, *NAALAD1* was found to plays a role in maintaining mechanical allodynia after carrageenan injection [28]. Of note, *NAALAD1* was also positively correlated with the DSPI but not to the same level of significance as *NAALAD2*. Finally, *NCALD* (neurocalcin delta) is a calcium-binding protein abundantly and almost exclusively expressed in the central nervous system that has not previously been associated with pain [29].

The genotyping study was conducted in samples obtained from twins enrolled in an ongoing independent IRB-approved pharmacogenomic study testing subjects' sensitivity to experimental heat and cold pressor pain among other outcomes (see Materials and Methods for details). The association study was performed using a generalized least square (GLS) test. GLS allowed us to model different variances between monozygotic twin pairs (MZ), dizygotic twin pairs (DZ), and the sexes, as each of these factors has previously been shown to influence pain measures [4], [30], [31], [32], [33].

**Figure 3:**
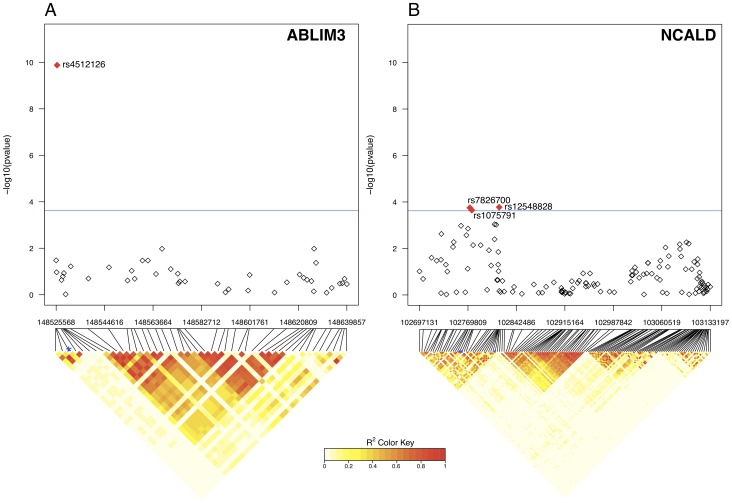
Manhattan plots and linkage disequilibrium heatmaps for *ABLIM3* and *NCALD*. Log10 transformed correlation association p-values with pain cold threshold for 43 and 132 SNPs located in the *ABLIM3* (A) and *NCALD* (B) genes regions, respectively. The x-axis represents the SNPs chromosomal physical location scale. The bottom heatmap represents linkage disequilibrium (LD) pairwise r^2^ based on the genotyped twin cohort. Blue star indicates polymorphisms found to be significantly associated with pain cold threshold.

**Figure 4:**
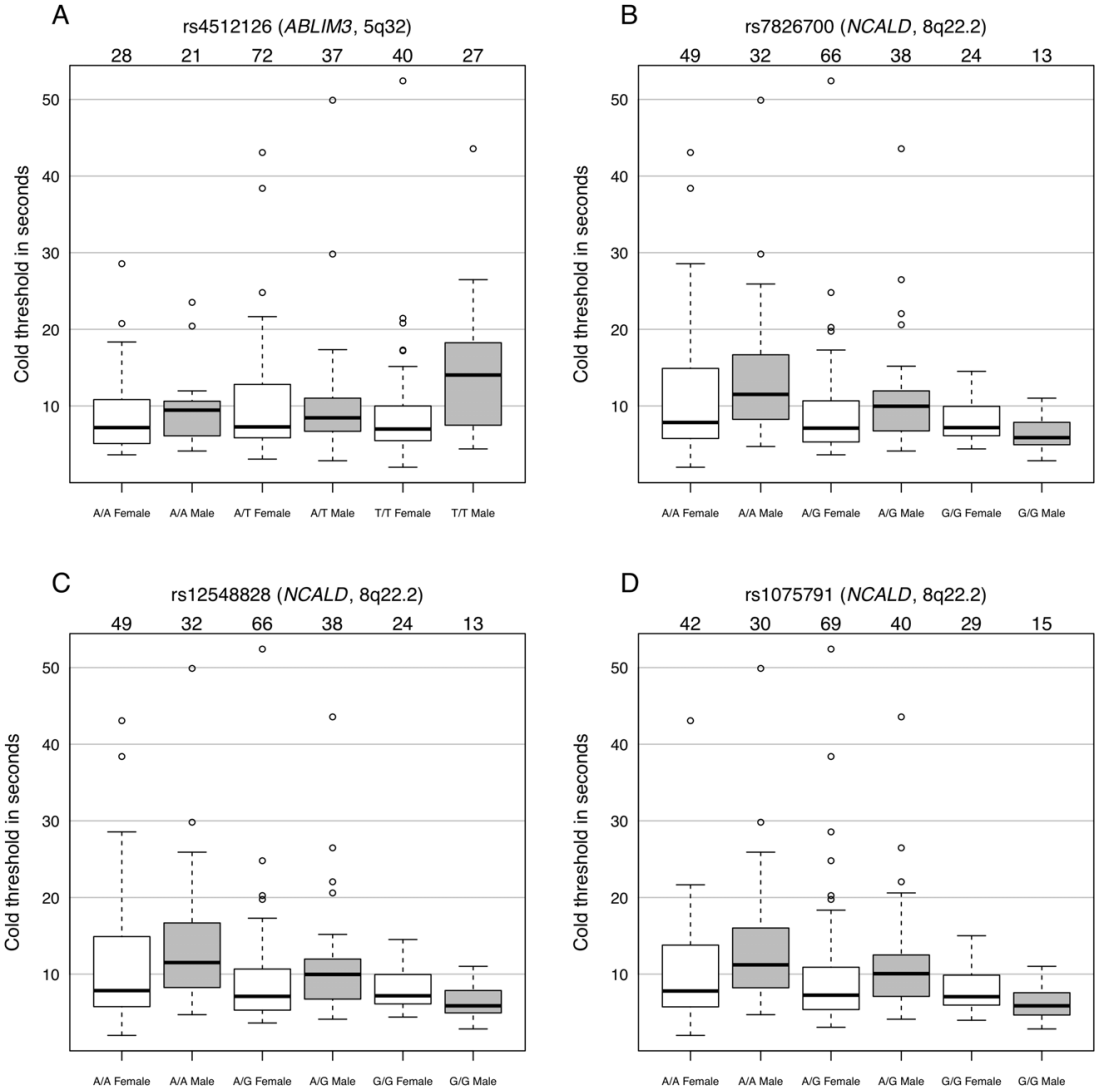
Boxplots of pain cold threshold by genotype and sex for polymorphisms found to be significantly associated with cold pressor pain. Dark lines within boxplots indicate the median. Box limits display first and third inter quartile. Horizontal lines at the limit of the boxplots represent the upper and lower end of the nominal data range defined as the upper quartile plus 1.5 times the inter quartile difference (IQD) and, the lower quartile minus 1.5 times the IQD, respectively. Open circles represent outliers falling outside the nominal data range. Numbers displayed above the boxplots indicate the number of individuals in the respective group.

Within the five selected genes, 251 tag SNPs were tested. Polymorphisms in *ABLIM3* (rs4512126) and *NCALD* (rs12548828, rs7826700, and rs1075791) showed significant association with the cold pressor pain threshold after Bonferroni correction (Figure 3A–B). Linkage disequilibrium (LD) analysis of the genotyped SNPs revealed a relatively weak LD structure around these polymorphisms. The LD structure in both genes was similar between the study cohort and the HapMap CEU population for the same region (Figure S2 and S3). Interestingly, the influence of the rs4512126 loci on the cold pressor pain threshold was tested, which revealed a male specific effect for individuals with the T/T allele (Figure 4A). Males with homozygous T/T alleles exhibited a significantly higher mean pain cold threshold than all other groups (p = 0.005, 4×10^−4^, 0.02, 0.005, 0.01, for A/A Males, A/A Females, A/T Male, A/T Females and T/T Females, respectively). The largest effect sizes (Cohen's *d*) were observed between T/T Males and A/A Males and Females (0.38 and 0.39, respectively). Effect sizes between T/T Males and the other groups were below the small effect size threshold (< = 0.2) with 0.16, 0.11 and 0.17 for A/T Males, A/T Females and TT Females respectively.

## Discussion

The primary objective of this study was to demonstrate the utility and validity of a novel, data-driven approach for generating a list of pain gene candidates. Such a list could facilitate the discovery of pain genes. We first validated our approach by demonstrating a statistically significant sensitivity and specificity prioritization of known pain-related genes contained in the Pain Gene Database (PGD). In addition, further genotyping of a human cohort revealed a significant association between variants of the newly discovered pain gene candidates *ABLIM3* and *NCALD* with measures of pain sensitivity in an independent human cohort.

A major emphasis of this study was to document the utility of the principal approach and highlight its future potential. The ever growing amount of publically available molecular and clinical data should allow for expanding and refining this approach to generate more comprehensive and specific lists. For example, as more data becomes available, it may be possible to link gene expression of diseases to specific types of pain, such as neuropathic pain. Similarly, the outlined approach can be expanded to include proteomic data sets, which should provide additional insight into signaling pathways relevant to the processing of pain. Finally, the pain associated with a specific disease can be construed differently. For example, disease-specific pain ratings could be retrieved from databases of large health care organizations [34].

There are a few limitations in our approach and study. First, among the 47 candidate pain genes significantly correlating with the DSPI, only two are referenced in the PGD. While the PGD is a valuable resource of curated information and likely represents the best available reference, it is not yet a globally accepted master repository containing all pain genes, especially those resulting from human studies. The database is constrained by the fact that it only catalogs genes revealed by studies examining nociception in mechanistic – but not disease-related – models in knock-out mice. It should also be noted that gene expression data for diseases and matched controls were only available for 121 diseases. As a result only 130 of the 300 genes listed in the PGD could be explored in the current study.

The presented paradigm did not capture genes such as *KCNS1*, *GCH1*, *COMT* or *OPRM1*, each of which has been implicated in the processing of pain [9]. This may partially be due to the fact that the current algorithm favored the discovery of genes exhibiting gradual gene expression change across different diseases. Additionally, our approach relied on gene expression changes in diseased tissue, which may not always capture important changes in secondary tissues relevant for the processing of pain, such as neuronal tissues or blood vessels. Additionally, some of these genes, such as *KCNS1*, are thought to be important in specific types of pain like neuropathic pain, but might not participate genetically in determining pain of other etiologies represented in the 121 diseases. There is considerable potential for more refined approaches in the near future to resolve some of these limitations, as there are a constantly growing number of publicly available repositories containing molecular and phenotypic data sets.

*ABLIM3* is a newly characterized protein-coding gene belonging to the actin binding LIM protein family, which is composed of 3 members (*ABLIM1-3*) and shows a high degree of conservation throughout evolution in vertebrates. *ABLIM3* is expressed in various tissues, most prominently in muscle and neuronal tissue [35], [36]. While relatively little is known about the biological function of the *ABLIM* protein family, conservation of key structural features suggests comparable biological function as linkers between the actin cytoskeleton, cell signaling pathways and transcription events [36]. For example, the ortholog of *ABLIM1* in *C. elegans* (UNC-115) has been implicated in axonal guidance during outgrowth through interaction with Receptor for Activated C Kinase (*RACK-1*) [37].

Presently, a potential functional role for *ABLIM3* in the perception or processing of pain is not apparent. *ABLIM3* could potentially affect nociceptive signaling by regulating synaptic strength through actin rearrangement and modulation of synaptic spine density [38]. Neuroplasticity has been shown to play a role in pathological pain and to happen both at the molecular and cellular levels [39]. However, the association of *ABLIM3* with pain is a novel finding that is based on a data-driven approach but is not anchored in our current understanding of pain biology. While this approach may offer the advantage of making unexpected and important discoveries, it requires establishing the biological relevance of such discoveries in subsequent experimental steps. The SNP rs4512126 (5q32) is located in the second and largest intron of *ABLIM3*. This variant was found in weak linkage disequilibrium (>0.6) with five other SNPs and in perfect linkage disequilibrium with rs4546368 located in the same intronic region of *ABLIM3*. All were non-coding SNPs. Similar to *ABLIM3*, *NCALD* has never been reported to be associated with pain. However, several polymorphisms in the 3′ UTR have been associated with mRNA instability and diabetic nephropathy [40]. Individuals carrying the A/A allele possessed a higher cold pain threshold. Nevertheless, we acknowledge that our discovered association between *NCALD* and pain cold was modest, demonstrated in only a single cohort, and barely above the Bonferroni corrected threshold. The parallel approach using quantile normalized phenotypical pain measures did not sustain the association for *NCALD* (Figure S4). Further genotyping in alternative cohorts and deep sequencing of these regions would be needed to reveal a potentially causal SNP.

Interestingly, only males with homozygous *ABLIM3* T/T showed a significant association with cold pressor pain sensitivity in our study. The sex-specific association of a gene variant with the cold pressor pain threshold is not surprising. Genetic polymorphisms associated with pain in humans and animals have identified a striking number of sexual dimorphisms with either male- or female-specific genetic effects, or a significant difference between the sexes [7], [9], [30], [34], [41].

We present a novel paradigm linking publically available molecular data to clinically relevant phenotypic data for generating a list of candidate genes relevant to the processing of pain. Algorithms for accessing and integrating such data to examine disease-relevant mechanisms are of growing interest as publically available data sets grow at an ever-increasing rate. The outlined approach can complement existing research efforts by assisting the formulation of data-driven hypotheses, and may serve as a template to discover genetic components of other clinically important phenotypes.

## Materials and Methods

### Ethics statement

The twin study was approved by the Institutional Review Boards of Stanford University and SRI International. All subjects gave written informed consent prior to participation.

### Linking disease to pain

MeSH is a comprehensive vocabulary thesaurus organized in a hierarchical structure allowing the indexing of publications with various levels of specificity. MeSH terms are used by trained human curators to annotate publications referenced in MEDLINE. We first built a thesaurus of 3743 disease-related MeSH terms using the Unified Medical Language System (UMLS); restricting ourselves to terms belonging to the following semantic type: *Pathologic Function* (T046), *Disease and Syndrome* (T047) and *Neoplastic Process* (T191) [42].

Searching MEDLINE for each of these MeSH terms gave us the number of publications published on each disease. For each disease returning a result, we conducted a second search in MEDLINE to count the number of papers published that were also annotated with the MeSH heading term “pain”[mh]. Searching MEDLINE for “pain”[mh] includes publications annotated with any terms hierarchically below “pain” in MeSH, such as *Aches*, *Burning pain*, and others. MEDLINE searches were automated using the EUtils programming tools available from the NCBI (http://eutils.ncbi.nlm.nih.gov). The ratio of these two counts formed the disease-pain ratio, as shown in equation (1).
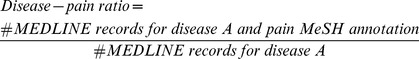
(1)


**Figure 5:**
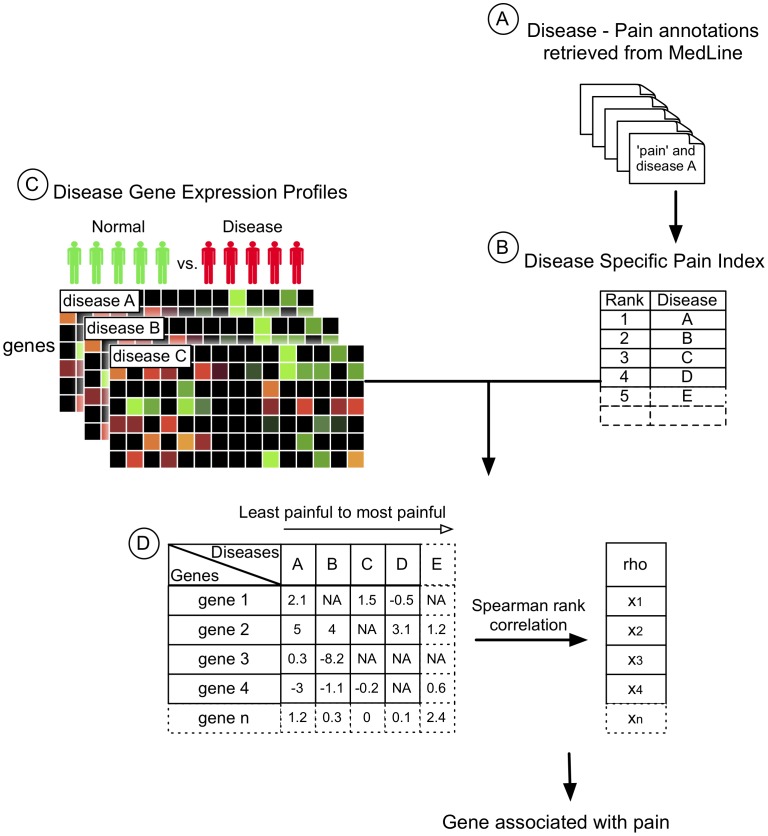
System-based approach to pain-gene candidate prioritization. (A) Publications annotated with the MeSH term “pain”[mh] in conjunction with a disease MeSH term were retrieved from MEDLINE. (B) The co-citation ratio (see Materials and Methods) established a disease-specific pain index (DSPI) representing the relative painfulness of a disease compared to others. (C) The disease-associated experiments publicly available from GEO and AE were retrieved and significantly differentially expressed genes were extracted in a disease-specific manner. (D) Disease gene lists were organized according to the DSPI from highest to lowest. The gene expression fold change was correlated with the disease ordering to determine significant associations between gene expression patterns and the pain index. Genes were ranked according to their Spearman rank correlation coefficient (rho) and p-values corrected for multiple hypothesis testing.

The comprehensive disease-specific pain index (DSPI) was established by ranking all 2962 diseases by their respective disease-pain ratio (Table S1). Implicit to our algorithm is the assumption that each disease provides unique qualitative information that may be diluted if weighting results by publication frequency. Thus, the only criteria for inclusion of the disease in the DSPI was to have at least one co-citation with a “pain” related MeSH term (Figure 5A–B).

A similar approach was followed to establish the Disease-Specific Inflammation Index (DSII) by searching for publication annotated with the MeSH term “Inflammation”[mh].

### Extracting disease associated gene expression profiles from public genomic repositories

We annotated all datasets from the National Center for Biotechnology Information (NCBI) Gene Expression Omnibus (GEO) and the European Bioinformatics Institute (EBI) ArrayExpress (AE) public databases with UMLS identifiers for diseases as previously published [43], [44], [45], [46], [47]. We further evaluated these data sets to determine whether or not the submitted biological experiments measured a normal control state (disease free tissues) complimentary to the annotated disease state. This was done to ensure that differentially expressed (DE) genes could be extracted for each disease. Drug treated samples were excluded from the study. Disease, tissue and substance annotations were manually reviewed in a post-processing step to ensure accuracy. Extraction of data from GEO and AE according to outlined steps revealed 311 diseases explored across 456 publicly available data sets and comprising 14,457 individual microarrays from 169 different tissues. In this study, microarrays were pre-processed and DE genes lists were generated using Rank Product (Figure 5C) [48]. We kept only genes with a q-value (gene-specific false-discovery rate) level ≤0.05 [49].

### Association of the DSPI with gene expression

One-hundred twenty-one of the 311 diseases retrieved from GEO and AE were also present in the DSPI list, and thus could be associated with disease-specific pain indices (see Table S1 for the DSPI and Table S2 for the overlapping list of 121 diseases). A small number of animal diseases were present in our DSPI since MEDLINE also covers veterinary and animal diseases. These were automatically excluded from the rest of the analysis, as retrieved gene expression data were limited to humans. Fold change values of the DE genes for each of 121 diseases were organized into a matrix with diseases as columns and genes as rows (Figure 5D). Because the lists of DE genes varied from one disease to another, we defined an arbitrary threshold of minimum gene representation. Information on a gene had to be present in at least 10% of the listed diseases. The final matrix contained 3812 genes as rows and 121 diseases arranged in columns. Finally, disease-columns in the matrix were ordered according to their DSPI rank, i.e., from the lowest to the highest pain index.

The Spearman rank correlation was then computed for each gene using the fold-change values against the DSPI ranking. We computed the positive false discovery rate (pFDR) values for each gene by permuting the diseases rank and re-computing the Spearman correlation for each gene. The operation was repeated 1000 times to obtain a null distribution of the correlation coefficients. The pFDR values were calculated as the ratio of the expected proportion of false positive *V* over the total number of hypothesis rejected *R* (2) [50].
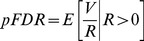
(2)


### Sensitivity and accuracy

We evaluated the sensitivity and accuracy of our method in prioritizing pain genes using Receiver Operating Characteristic (ROC) curves. We compared the DSPI-based pain-gene list against a list of known pain genes from the PGD (www.jbldesign.com/jmogil/; accessed October 2010) [18]. We acknowledge that the PGD only contains data from studies in knockout mice. However, the PGD is to our knowledge the only available repository of pain genes. All 308 mouse genes in the PGD were retrieved manually and translated to 300 human homologs using the NCBI Homologene database (www.ncbi.nlm.nih.gov/homologene). All 300 mouse genes were translated to unique human genes except 8 genes lacking homologs. These eight genes were not further considered. We established the confidence intervals for the ROC curve using a leave-one-out resampling method by repeatedly recalculating the pain-gene rankings with nine-tenths of the 121 diseases. These alternative pain-gene lists were then used to compute the confidence interval of the standard error of our pain-gene ranking. We used the ROCR package from R to compute the ROC curves [51]. Of note, the gene expression measurements on the 121 diseases only included 130 of the 300 known pain genes from the PGD.

Gene extracted using the DSII were compared to a gold standard made from the genes belonging to the “Inflammatory response” Gene Ontology category (GO:0006954). The gene list was retrieved from the Molecular Signature Database [52].

### Twin cohort and experimental pain measurements

Our study used samples and data from a pre-existing large pharmacogenomic study in twins examining the heritability of various opioid effects [32]. More specifically, data on subjects' sensitivity to heat and cold pressor pain before drug exposure were retrieved. The twin study was approved by the Institutional Review Boards of Stanford University and SRI International. All subjects gave written informed consent prior to participation.

In total, 228 healthy pain-free twins (114 twin pairs) of diverse ethnicities were genotyped and phenotyped. We considered only individuals with self-declared European ancestry, which represented 179 individuals with an age range of 18–68 years. Zygosity status of identical (MZ) and fraternal (DZ) twins was assessed by genotyping concordance using a panel of 47 SNPs [53]. Relevant covariates known to potentially confound measures of pain were also assessed and included demographic factors, age, sex, education, depressed mood, anxiety, sleep, and blood pressure. A detailed description of methods has been published elsewhere [32]. Overall, the cohort used for this analysis was not balanced for sex (107 females and 72 males) and consisted of 54 dizygotic and 125 monozygotic twins.

Experimental pain measurements were performed in the Human Pain Laboratory of the Department of Anesthesia at Stanford University School of Medicine. Heat pain was induced with a thermal sensory analyzer (TSA-II, Medoc Advance Medical System, Durham, North Carolina). A 3×3 cm thermode was placed in contact with skin at the volar forearm. Starting at 35°C, the thermode temperature was increased at a rate of 1°C/s. Study participants pushed a button of a hand-held device at the onset of pain. This procedure was repeated 4 times with an inter-stimulus interval of 30 seconds. The average temperature (C°) eliciting pain was recorded as the pain threshold.

Cold pressor pain is thought to mimic important qualities of clinical pain, since verbal descriptors for both types of pain are strikingly similar [54]. Cold pressor pain is more sustained than heat pain and is associated with a much stronger affective response [55]. The cold-pressor pain model can be viewed as a tool examining an integrated pain response with a strong affective component, while the heat pain model is better suited to explore sensory-discriminative aspects of pain. Sensitivity to cold pressor pain was tested by having subjects immerse their hand to mid-forearm in ice-water (1–2°C) continuously recirculated within a 12-liter container. The palm of the hand was in full contact with the bottom of the container. Subjects were asked to indicate the onset of dull, aching pain typically perceived in the wrist and to withdraw the hand once pain became intolerable. The time (seconds) to the onset of pain was recorded as the cold pain threshold and the time to withdrawing the hand was recorded as the cold pain tolerance.

### Genetic association study

The twin cohort was genotyped for 251 SNPs across 5 genes selected from the list of candidate pain genes (Table 2). We selected LD tag SNPs (r^2^ = 1) based on the HapMap CEU population using the Tagger software from the Broad Institute (http://www.broadinstitute.org/mpg/tagger/) [56]. Twin's DNA was extracted from peripheral blood lymphocytes and genotyped using a custom-designed Oligo Pool for Methylation Assay (Golden Gate Genotyping Assay, Illumina, Inc, San Diego, CA) and BeadXpress (iGenix Inc., Bainbridge Island, WA). We filtered out all SNPs with a call rate <90%.

Out of 251 SNPs assayed only 216 yielded successful results following Illumina quality controls. Some successfully genotyped loci had missing genotypes for a small number of twins and were imputed using the homozygous wildtype allele from the population. We filtered out all SNPs with a minor allele frequency <5% or those whose genotype frequency departed from Hardy-Weinberg equilibrium at p<0.01. In summary, 207 SNPs were tested against heat pain threshold, cold pain threshold and cold pain tolerance.

While twin individuals were not required to test association of our candidate genes with pain, twins allowed us to control for environmental variability in pain measurements. Rather than utilize methods to correct for the relatedness of observations coming from the two members of the same twin pair, we used a model in which each pair was treated as a single observation by using within-pair genotype and phenotype averages. Genotypes were transformed to numbers according to the allele frequency, 0, 1, 2 for homozygous wildtype, heterozygous and homozygous rare, respectively. Then, the genotypes of twins were averaged within each pair. The genotype of single twin was not altered. Categorical covariate data such as sexes were discretized to −1 and 1 for male and female respectively and then averaged. DZ twin pairs of different sex were coded 0.

To test association of measures of pain sensitivity with each SNP, we used a generalized least square (GLS) regression model [57] examining the null hypothesis that pain and genotype are not associated. We regressed on the pain score measured, *y*, against the genotype for the SNP considered while controlling for depression of the individual (Beck Depression Index) and sex (3).
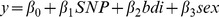
(3)


GLS allowed us to model different variances of measured traits in MZ and DZ twins and in males and females. We related genotype to the heat pain threshold (degree C°) and the log of both the cold pressor pain threshold and cold pressor pain tolerance. P-values were corrected for multiple hypotheses testing using the Bonferroni correction. One twin with the T/T allele for rs4512126 reached the maximum allowed time in the cold threshold test (3 min). This result was considered an outlier and was removed from the analysis. In parallel, we also evaluated the effect of the pain phenotype normally distributed through quantile normalization. This analysis revealed similar results. The rs4512126 (*ABLIM3*) polymorphism remained significant for cold pain threshold (Figure S4A). Additionally, rs4512126 and rs7715362 (*ABLIM3*) showed a significant association with cold pain tolerance (Figure S4B) and rs7720260 (*ABLIM3*) showed a significant association with heat pain threshold (Figure S4C). However, previously found significant polymorphisms in *NCALD* did not pass the Bonferroni threshold in this analysis.

We computed the Cohen's *d* effect sizes for the difference observed between men and women homozygous for the minor allele of the rs4512126 variant as follows *d* = [*x_m_*−*x_f_*]/*pooled standard deviation*, where *x_m_* and *x_f_* are the average cold pressor pain threshold in males and females, respectively [58]. Positive values indicate a higher male average pain threshold, and negative values indicate a higher female average pain threshold. The result is unit free and Cohen proposed that benchmark values for what should be considered a ‘small’, ‘medium’ and ‘large’ effect (*d*> = 0.2, 0.5, 0.8, respectively) [59]. We analyzed differences in mean cold threshold for rs4512126 by performing two-sample *t*-tests with unequal sample size and unequal variances (Welch two sample *t*-test).

## Supporting Information

Figure S1 **Receiver Operating Characteristic (ROC) curve.** The ROC curve depicts the performance of our algorithm to identify known inflammatory genes belonging to the Gene Ontology “Inflammatory response” category. The area under the ROC curve is 73.2%, which is significantly different from random chance.
(TIF)

Figure S2 **Linkage Disequilibrium comparative representation of the** ***ABLIM3*** **SNPs.** (A) LD structure (r^2^) of genotyped SNPs within our twin cohort. (B) LD structure of the same SNPs using HapMap II+III population with European ancestry. Blue star indicates rs4512126 SNP location.
(TIF)

Figure S3 **Linkage Disequilibrium comparative representation of the** ***NCALD*** **SNPs.** (A) LD structure (r^2^) of genotyped SNPs within our twin cohort. (B) LD structure of the same SNPs using HapMap II+III population with European ancestry. Blue star indicates rs12548828, rs7826700 and rs1075791 SNPs location.
(TIF)

Figure S4 **Manhattan plot for** ***ABLIM3*** **polymorphisms when pain phenotype are quantile normalized.** (A) Represent association p-values for SNPs in *ABLIM3* with cold pain pressor threshold. (B) Association of *ABLIM3* polymorphisms with cold pain tolerance. (C) Association analysis of *ABLIM3* polymorphisms with heat pain threshold.
(TIF)

Table S1 **Disease-specific pain index (DSPI) ranking all 2962 diseases by their respective disease-pain ratio.**
(XLS)

Table S2 **List of 121 diseases retrieved from GEO and AE that were also present in the DSPI.**
(XLS)
