# Supplementary figures and images for: Integrative Approach to Pain Genetics Identifies Pain Sensitivity Loci across Diseases
Source: PLoS Comput Biol. 2012 Jun 7;8(6):e1002538. doi: 10.1371/journal.pcbi.1002538 (PMC3369906; doi:10.1371/journal.pcbi.1002538)

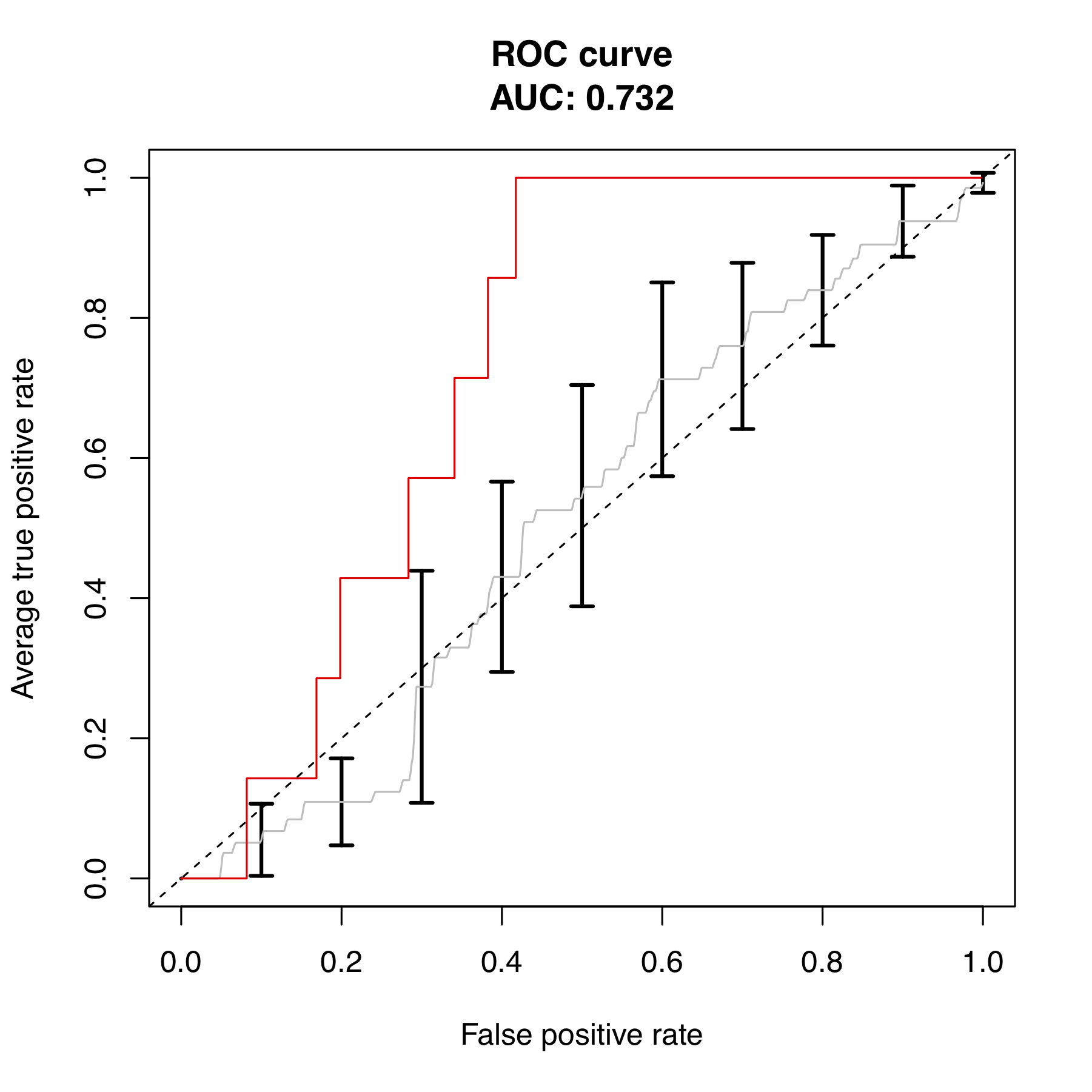

Supplement: Figure S1 — Receiver Operating Characteristic (ROC) curve. The ROC curve depicts the performance of our algorithm to identify known inflammatory genes belonging to the Gene Ontology “Inflammatory response” category. The area under the ROC curve is 73.2%, which is significantly different from random chance. (TIF) [file pcbi.1002538.s001.tif]

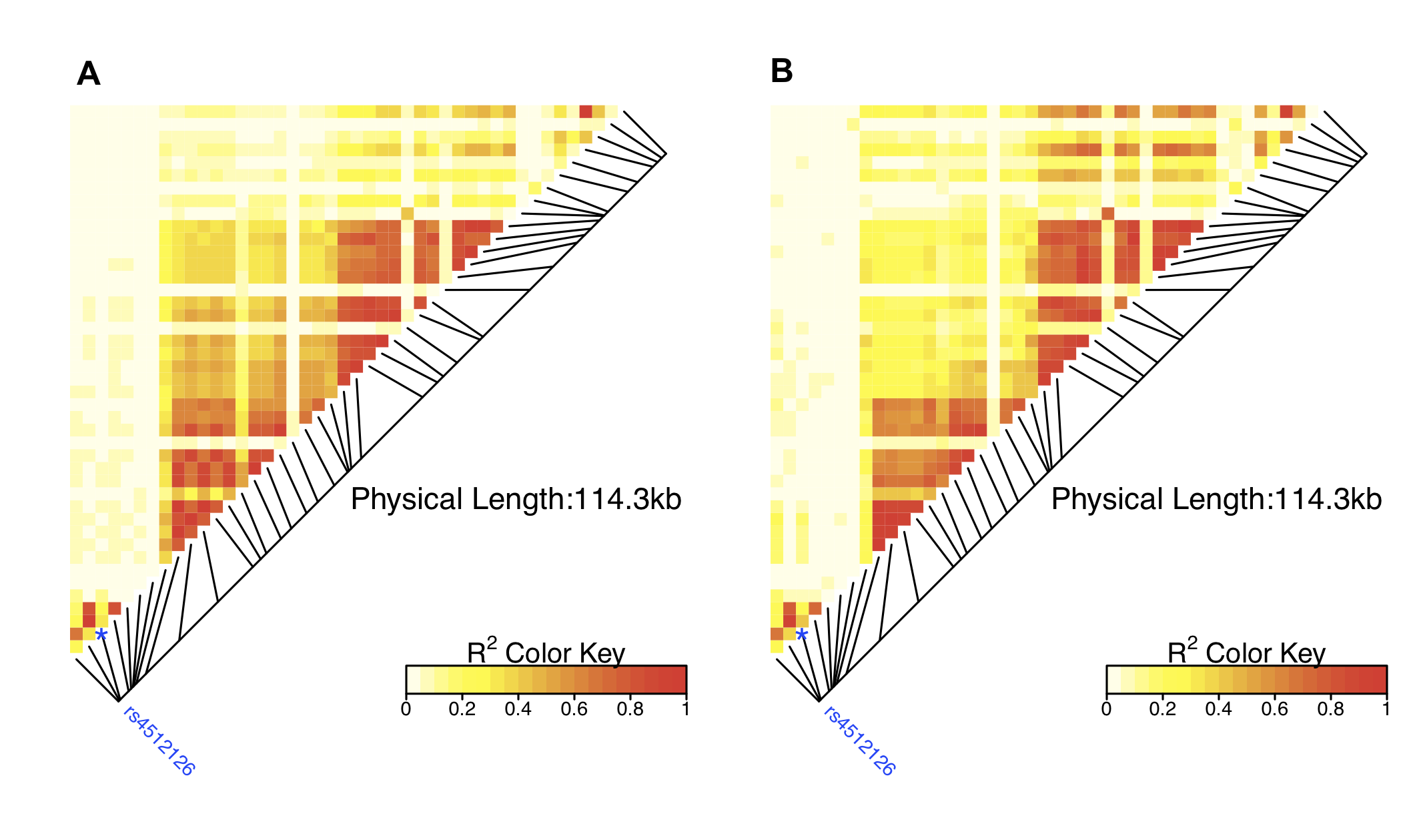

Supplement: Figure S2 — Linkage Disequilibrium comparative representation of the ABLIM3 SNPs. (A) LD structure (r2) of genotyped SNPs within our twin cohort. (B) LD structure of the same SNPs using HapMap II+III population with European ancestry. Blue star indicates rs4512126 SNP location. (TIF) [file pcbi.1002538.s002.tif]

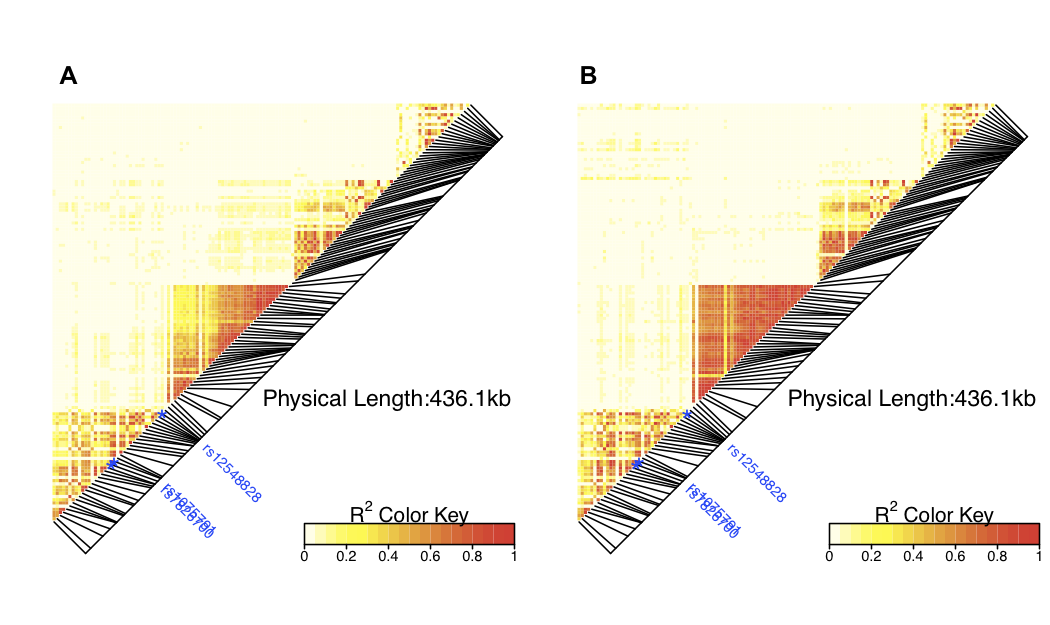

Supplement: Figure S3 — Linkage Disequilibrium comparative representation of the NCALD SNPs. (A) LD structure (r2) of genotyped SNPs within our twin cohort. (B) LD structure of the same SNPs using HapMap II+III population with European ancestry. Blue star indicates rs12548828, rs7826700 and rs1075791 SNPs location. (TIF) [file pcbi.1002538.s003.tif]

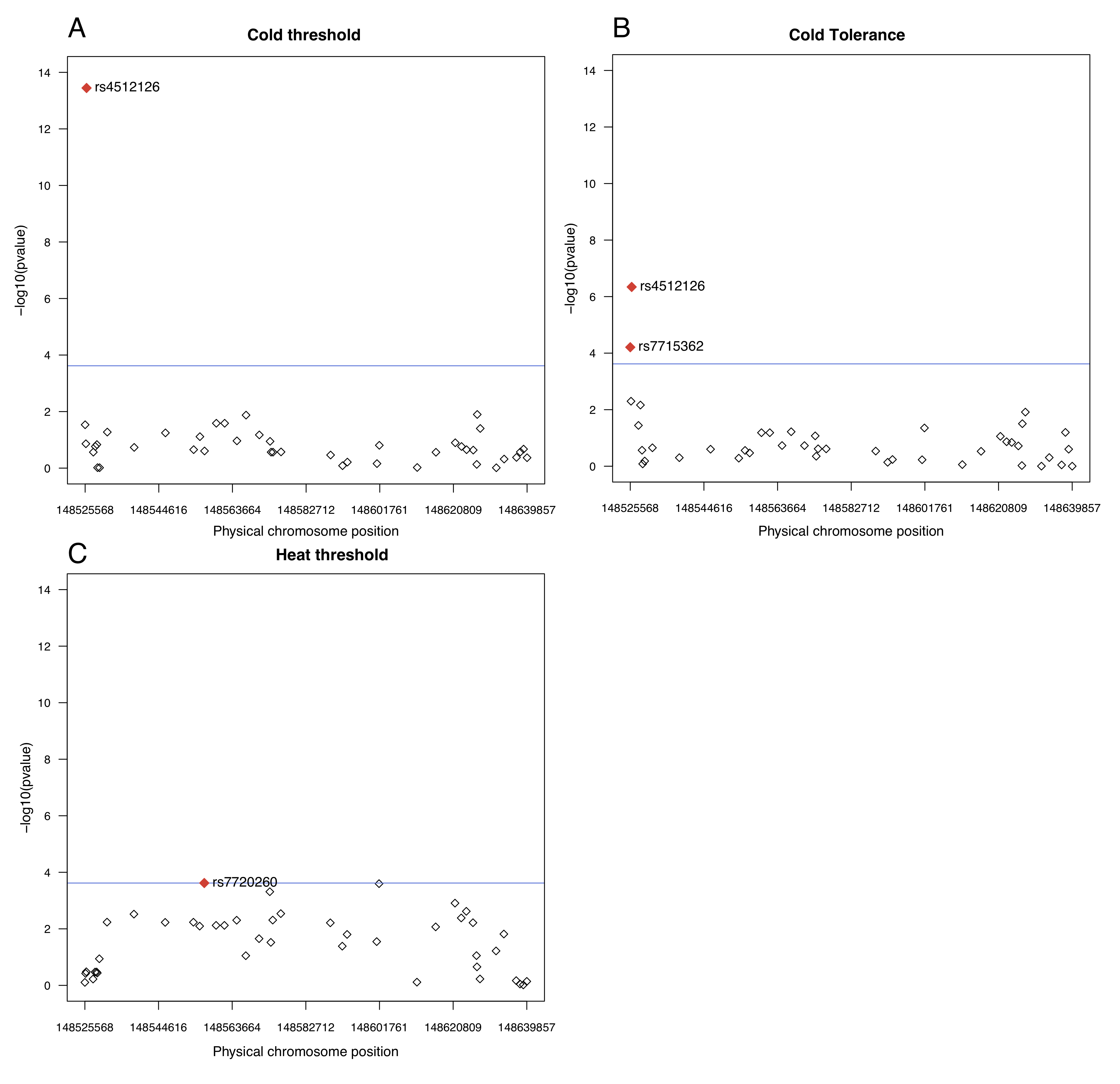

Supplement: Figure S4 — Manhattan plot for ABLIM3 polymorphisms when pain phenotype are quantile normalized. (A) Represent association p-values for SNPs in ABLIM3 with cold pain pressor threshold. (B) Association of ABLIM3 polymorphisms with cold pain tolerance. (C) Association analysis of ABLIM3 polymorphisms with heat pain threshold. (TIF) [file pcbi.1002538.s004.tif]
